# The Natural History of Bone Stress Injuries in Athletes: From Inception to Resolution

**DOI:** 10.1007/s40279-025-02280-9

**Published:** 2025-07-28

**Authors:** Melissa L. Crunkhorn, Naroa Etxebarria, Liam A. Toohey, Paula Charlton, Kate Watson, Michael Drew

**Affiliations:** 1 University of Canberra Research Institute for Sport and Exercise (UCRISE), Canberra, Australia; 2 Queensland Academy of Sport, Brisbane, Australia; 3 Aus Triathlon, Gold Coast, Australia; 4 Australian Institute of Sport, Canberra, Australia

## Abstract

Bone stress injury (BSI) occurrence is common in athletic populations, resulting in high periods of time loss from sports participation. Minimising incidence and reducing severity presents a challenge for the prevention and clinical management of bone stress injuries (BSIs) for sports practitioners. An understanding of the aetiology and mechanisms for BSIs in athletic populations can assist with the design and implementation of prevention programmes. Application of established health frameworks allows practitioners to identify and manage the complex and dynamic interplay of factors that alter the susceptibility of an athlete to the onset, and subsequent progression of BSIs. The natural history of disease describes well-defined sequential stages of disease progression, sequenced from pathological onset through to disease outcome that occurs in the absence of clinical intervention. The purpose of this review is to synthesise and map the current evidence on BSIs to the natural history of disease. This review will provide sports medicine practitioners with a clinically applied framework that aligns current evidence to stages of disease, with reference to intervention and management. In addition, targeted prevention strategies are described and mapped to primary, secondary and tertiary levels of prevention along the BSI continuum. Despite the extensive body of evidence detailing BSIs in sport, this paper is the first to integrate and map BSIs to the natural history of disease.

## Key Points

**Table:** 

The prevention and clinical management of BSIs is challenging, and the burden of these injuries has a substantial impact on athletes, sports participation, sporting organisations and health systems.
The development and application of an epidemiological framework of BSIs in athletic populations may assist in optimising the most effective preventative strategies.
Targeted prevention strategies should address the primary, secondary and tertiary prevention phases along the bone stress injury continuum, focusing on specific athlete subgroups and risk factors for the development of BSIs.

## Introduction

Bone stress injuries (BSIs) commonly occur in athletic populations and negatively impact athletes’ immediate and long-term health. Bone stress injuries result in substantial periods of time-loss from sport, limit training and can be the catalyst for premature retirement and life-long consequences [1]. The prevention and clinical management of BSIs is challenging, and the burden of these injuries has a substantial impact on athletes, sports participation, sporting organisations and health systems [2]. Whilst the pathophysiology of BSIs is extensively documented, and current clinical management strategies aim to detect these injuries as early as possible; the application of an epidemiological framework could assist with how the management of BSIs can be optimised through a systems lens.

The purpose of this review is to synthesise the current evidence on BSIs relative to the stages of the natural history of disease (an accepted clinical epidemiological framework), with reference to intervention and management, and evidence has been sought to support the use of this framework in the disease state. This review discusses targeted prevention strategies to address the primary, secondary and tertiary prevention phases along the BSI continuum, focusing on specific athlete subgroups and risk factors for developing BSIs. The application of an epidemiological framework for BSIs in athletic populations may assist in optimising the development of targeted effective preventative strategies.

## Epidemiology of Bone Stress Injuries

### Incidence and Prevalence

There are limited recent epidemiological data on BSIs in athletic populations and the incidence of BSIs varies according to sport. Sports that involve high impulse forces and repetitive cyclical bone loading (mechanical load), where athletes are exposed to high training volumes (i.e. athletics, gymnastics, basketball and rowing) have the highest reported incidence of BSIs [3–8]. There is a high recurrence rate reported for BSIs, with recurrence rates of lumbar spine BSIs of 39% in cricket, and also one study reporting 15% of athletes across a variety of sports reporting two or more BSIs during the 3-year surveillance period [3, 9]. Bone stress injuries have been reported to account for 15–20% of all musculoskeletal injuries in cross-country, track and field, triathlon, duathlon and recreational running athletes [10]. The incidence of BSIs in American high school and National Collegiate Athletic Association athletes is 1.5 and 5.7 BSIs occurring per 100,000 athlete-exposures, respectively [5, 6]. In a retrospective clinical audit in the Australian High Performance System, including 48 different sports over a 3-year period, the majority of BSIs were reported during training (66%) as opposed to competition (7%) [3]. However, the proportion of BSIs within athlete cohorts in this study ranged widely among sports.

### Distribution

#### Body Site

The body sites of BSI occurrence vary from sport to sport. The exact location of BSIs is influenced by how the skeleton is loaded, with the majority of BSIs occurring in highly loaded bones (e.g. lower extremities) [7]. Most BSIs in endurance runners occur in the lower limb (80–95%), mainly in the tibia (49%) and tarsal bones (25%) [8, 11–14]. These injuries are often related to running, which is also evident in multidisciplinary endurance sports such as triathlon [15, 16]. The majority of BSIs in gymnasts and fast bowlers in cricket occur in the lumbar spine [3, 17] and in the ribs in rowing athletes [3, 4], due to the extremes in trunk range of motion, coupled with the application of cumulative mechanical loads. Although more uncommon owing to the non-weightbearing nature of the upper limbs, BSIs can occur in the arm, primarily in the humerus and ulna in tennis players and pitchers in baseball and softball, where the upper limb is exposed to excessive cyclical force through chronic torsional stress [18, 19].

#### Sex Differences

Bone stress injury occurrence varies according to sex, where females appear to have a higher incidence in both athletic and military populations [1]. Female runners are reported to have a BSI incidence rate 1.8–2.3 times higher than male runners [5, 6, 10, 20–22]. Similarly, female endurance runners and track and field athletes have 10% and 7% higher rates of BSIs than their respective male counterparts [1, 23, 24]. In female triathletes, a 2.9-fold higher rate of BSIs has been reported compared with male triathletes [15]. Female-specific physiological and biomechanical factors that influence bone health are detailed extensively in literature [7, 20, 25–27], and these sex-based differences indicate that BSIs affect males and females differently, which is why sex must be considered when understanding the epidemiology of BSIs [28].

#### Exercise Volume/Competition Level

Repeated, cumulative exposure to mechanical load in activities such as running and jumping, as well as new or extreme exercise patterns can predispose individuals to BSIs [29]. There is no consensus on a threshold of exercise volume at which BSIs are likely to occur, as all athletes and sports are inherently different, however, athletes completing a higher number of training hours per week are more likely to develop BSIs [26]. Some studies report female adolescent athletes training between 12 and 16 h or more per week are at an increased risk of BSIs, as are male adolescents running greater than 30 miles a week [30–32]. These training load figures are substantially lower compared with typical training loads of elite athletes [33, 34]. Determining an exact relationship between level of sport, weekly training load and BSIs is highly individualised and multifactorial, including the time-varying nature of individual exposures to these factors.

## Natural History of Disease

### Overview—Stages of Disease

**Fig. 1:**
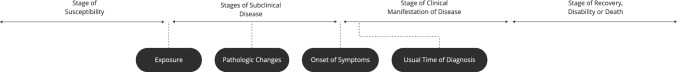
Natural history of disease (adapted from the Centers for Disease Control and Prevention) [37]

The natural history of disease is a well-established framework applied to many medical conditions (e.g. diabetes and cancer), detailing the course of a specific disease in the absence of clinical intervention from pathological onset (inception) through to disease outcome (complete recovery, morbidity or permanent impairment or eventual death) [35, 36]. The three stages are: (1) susceptibility, where the condition has not yet developed, but an individual has been exposed to factors that increase the risk for the condition to develop; (2) sub-clinical stage, which is the time period from the initiation of the condition to the first appearance of signs and symptoms; and (3) clinical manifestation of disease, where signs and symptoms are present, and the disease may resolve, be subject to recurrences or progress to a fatal outcome (Fig. 1) [35–37]. These three stages are referred to throughout this review as they are the most clinically aligned, however, we note that primordial and quaternary stages also exist. These stages are out of the scope of this review, as they primarily focus on social and economic determinants of health (primordial stage), and activities to reduce consequences of unnecessary intervention (quaternary stage) [36]. Understanding the natural history of specific pathologies is critical, as early detection and intervention can alter the natural history of the condition [35]. When the natural history is intervened upon, the new progression of disease is termed the ‘clinical course’. Each of the above defined three stages of the natural history of disease for BSIs in athletic populations will be subsequently explained in detail.

### Stage of Susceptibility

#### Risk Factors for Bone Stress Injuries 

The aetiology of BSIs is multifactorial, where development follows exposure to a combination of interacting intrinsic and extrinsic factors [38–40]. Athletes are susceptible to BSIs as a result of the interaction of sets of risk and casual factors. Some of these factors are non-modifiable (e.g. age), and some are modifiable (e.g. training load) [41, 42]. While exposure to training and competition is required for a BSI to occur, there is no universal evidence across sports (with the exception of cricket) [43] around the quantification of exposure that leads to a BSI. Through understanding occupational risks (e.g. fast bowling versus spin bowling in cricket), there is evidence highlighting that certain positions have causal exposures (workload) which explains the biomechanical strain placed on the body [43]. Predisposed athletes can then become exposed to extrinsic risk factors (e.g. playing surface), making them susceptible to injury, which can be mediated by the response (adaptations or maladaptations) that occur with continued training exposure [41, 44]. Following this exposure, injury can arise from an inciting event (e.g. traumatic event) or cumulative tissue overload where biomechanical stress exceeds the ability of the athlete’s tissues to withstand the force, in which these forces are generated by an athlete’s exposure to sports activities (training and competition) or repetitive mechanical bone loading where microdamage accumulates and bone homeostasis is no longer able to be maintained [41]. To complement the model of prevention that is based on pathology, risk factor-based preventative strategies (universal, selective and indicated measures) are applied to specific populations participating in sport that display particular risk indicators that increase their susceptibility to injury [2]. Where it is possible to address risk factors at a whole-of-population level or where the risk is universally indicated, universal measures are recommended for anyone participating in sport [2]. Selective measures are targeted to sub-groups participating in sports characterised by age, sex, experience or other sports-related risk indicators [2]. Indicated measures are targeted to individuals considered ‘at-risk’, based on a risk indicator that infers increased injury susceptibility [2].

Proposed universal risk factors for BSIs include training surfaces, type of sport (team or individual), seasonality, footwear, exposure to high or low impact activities and repetitive loading [45, 46]. Gymnastics and athletics (running and jumping disciplines) are high impact sports where a high incidence of BSIs are reported, with increased risk during periods of adolescent growth [3, 47]. There is limited evidence to support footwear as a risk factor [46].

The following characteristics increase the susceptibility of sustaining a BSI in sub-groups of athletic populations according to studies exploring risk factors for BSIs: female sex, competitive sport participation, problematic low energy availability (LEA), vitamin D or iron deficiency, irregular menstrual cycles and training prescription errors [41, 48–50]. It is widely reported that female athletes have a higher incidence of BSI compared with male athletes [5, 29, 51]. Iron deficiency is prevalent in elite athletes, particularly females [52] and those with lower dietary iron intake or malabsorption issues. In addition, low levels of vitamin D are thought to increase susceptibility to sub-optimal bone health and muscle function [53, 54]. Menstrual status and age of menarche are also reported risk factors for sustaining a BSI. Menarche occurring after the age of 15 years is associated with lower bone mineral density, and a four-fold increased risk of stress fractures in adolescent female runners [27, 55]. Evidence suggests that low bone mineral density and bone mineral content are associated with a higher incidence and greater severity of stress fractures, with a consequent delayed return to sport [56]. Determining an exact relationship between weekly training exposure (type, frequency, duration, intensity) and BSI risk is highly individualised, dependent on many intrinsic factors and considerations. However increases in training volume and/or intensity have an impact on BSI risk, at least in females [31]. The degree by which training volume and intensity contribute to the risk of BSIs differs between individuals and requires further investigation as there is limited evidence assessing this complex (and likely causal) interaction.

The female athlete triad is one of the main factors reported to impact bone health in female athletes. The female athlete triad is the combination of disordered eating and irregular menstrual cycles that eventually lead to hormonal alterations including decreased endogenous oestrogen, which can result in low bone mineral density [57]. The triad has been refined to consider the occurrence of LEA in either the presence of absence of disordered eating, functional hypothalamic amenorrhoea and osteoporosis [58]. This definition better reflects the continuum of the three inter-related components ranging from a healthy endpoint to sub-clinical and clinical conditions [58]. Female athletes often present with one or more of the triad components and early intervention is critical to prevent progression to serious outcomes including clinical eating disorders, amenorrhoea and osteoporosis [59]. Delayed menarche [55], a history of menstrual disturbances [31, 56, 60], and/or low bone mineral density are all risk factors for BSIs in female athletes [31, 56, 61] and military recruits [62, 63]. Low energy availability has a causal role in the onset of menstrual disturbances resulting from exercise, which increases the susceptibility of female athletes for BSIs [64]. Mild-to-moderate low bone mineral density is present in female athletes with oligomenorrhoea and sub-clinical menstrual disturbances [65]. Given the impact that the female athlete triad has on bone health it is important to understand the factors that increase susceptibility of female athletes for BSIs.

Whilst there are a number of factors that increase susceptibility of an individual for a BSI, there are some protective factors which act to promote bone health and move an individual away from biological onset [66]. Certain modifying variables including optimising pre-pubertal bone mass [67], ensuring exposure to multidirectional sports before puberty [27, 68], avoiding early specialisation [68] and adequate energy intake [67] are all factors throughout an individual’s life course that can decrease their susceptibility to BSIs. Adolescent athletes with a history of participation in ball sports usually have higher than average bone mass, and appear to be protected from BSIs in future sports participation [27, 69]. Participation in multidirectional sports and cross training activities allows individuals to develop a range of motor skills and exposes them to a variety of multidirectional osteogenic stimuli [32, 68, 70]. Whilst these factors provide numerous health benefits and theoretically protect against BSIs, direct evidence is required [71].

#### Causal Model

**Fig. 2:**
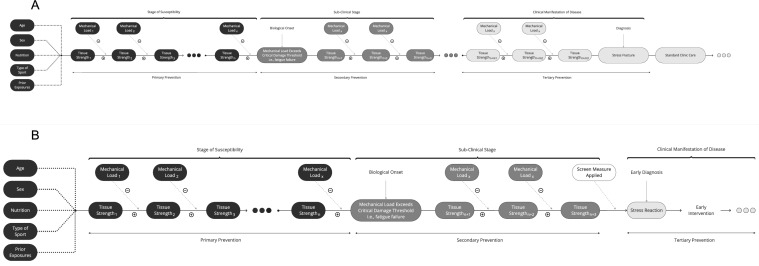
**a** Usual clinical course. **b** Early intervention model

Current sports injury aetiology models have investigated how the various BSI risk factors interact to increase injury risk in susceptible athletes [41, 44, 72, 73]. The various causes of BSIs can be considered conceptually as two fundamental components: (1) mechanical loading (referred to as bone loading), which is the force experienced by the bone; and (2) mechanical strength of the bone [74]. All casual variables must act through one or both of these factors. Figure 2 illustrates this proposed causal pathway for gradual onset injuries like BSIs from repetitive loads in athletes [74]. This figure outlines how repetitive mechanical loads cause fatigue in bone tissue until the critical damage threshold is exceeded and a BSI occurs [74]. In BSIs, it is also acknowledged that physiological processes have an important role in influencing tissue strength through bone remodelling [74], omitted from the figure for simplicity. In BSIs, the mechanical strength of the bone deteriorates over time. With repeated exposure to cyclic loading, bone strength differs after each instance of mechanical load application, and thus is represented by a new separate variable [74].

Owing to the multifactorial nature of injuries, single risk factors have limited predictive ability [72]. The casual pathway between training load, tissue damage and injury is unclear, with a definitive aetiology yet to be established [75]. While having limitations, the Rothman ‘sufficient-component cause model’ provides an accessible framework to conceptualise causality in a clinical context, where outcomes (disease/injuries) result from sufficient causes (risk factors) [76]. Each sufficient cause can be divided up into a ‘causal pie’, made up of ‘component causes’ [76]. This model recognises that diseases have multiple contributing factors that interact with each other to produce a given outcome [77]. A sufficient cause is not a single factor, but a minimum set of factors and/or criteria that, if present in a particular individual, will result in the disease/outcome [76]. Each component that makes up a sufficient cause is called a component cause [77]. For BSIs to occur, a number of precipitating factors must be present at the same time to contribute to the minimum causal set (sufficient cause). The set of factors leading to the onset of a BSI in one individual may not be the same factors that lead to BSI occurrence in others [76]. We acknowledge these are overly simplistic frameworks and that real-world models would require further complexity to be considered.

There are many possible sufficient causes for BSIs that consist of various component causes. A component cause that must be present in every sufficient cause for a given outcome is a necessary cause [77]. Exposure to mechanical load resulting in internal stress and strain in a manner that exceeds the biological ability of the bone to adapt to the force is a component cause, as well as a likely necessary cause in the development of BSIs. Bones will typically adapt to the degree of mechanical loading they are exposed to, however, the duration, magnitude, and rate of forces applied to the bone influence bone remodelling and homeostasis [78]. Using the model in Fig. 2 and applying it to the Rothman framework, the necessary cause for BSIs is mechanical loading, and it is the exposure to cumulative mechanical load that exceeds the biological capacity for the bone to adapt. In BSIs, it is important to acknowledge that exposure to a certain threshold of acute mechanical load is not necessary in isolation, as it is not cumulative. For BSIs to develop, cumulative exposure to the stresses and strain that occur with the application of force is likely the main necessary factor that needs to be present in every sufficient cause. All other factors are component causes with varying lead times [79]. With inadequate time to adapt between exposure to the necessary cause, damage accumulates owing to the positive feedforward loop of remodelling and damage formation [80]. Microdamage accumulation may coalesce, and this is where the BSI pathology continuum commences, beginning with stress reactions, followed by stress fractures progressing through to complete fractures [80]. Current evidence highlights that BSIs follow a cumulative causal model, as represented by Kalkhoven [74], which indicates that this cumulative exposure causal model is a necessary cause in all BSIs. Individual risk factors, and the affected bone differ for all BSIs, and are influenced by other characteristics.

#### Primary Prevention (Prevention Within the Susceptible Stage)

Primary prevention focusses on removing or reducing the causal factors for BSIs through health promotion [81]. Primary prevention should adopt a multifaceted approach and is typically delivered at a population or cohort level. Examples include promotion and encouragement of adoption of healthy behaviours, and the creation of a proactive safe environment that supports positive body image [50, 82]. One of the factors reported to impact athlete bone health is the female athlete triad. Primary prevention should focus on a multidisciplinary approach to increasing knowledge of the sequelae of problematic LEA pertaining to health and performance and increasing awareness around adequate energy availability. These strategies should focus on specific short-term education programs highlighting factors associated with the female athlete triad, eating disorders and disordered eating behaviours [59, 83]. There is evidence supporting the efficacy of education programs in improving nutritional knowledge and reducing signs of body image concerns and dieting behaviours in male and female athletes [84, 85]. Interactive workshops in male and female collegiate athletes and female dancers have effectively promoted positive body image, encouraged self-care, and decreased risk factors for eating disorders [50]. Optimising energy availability through education and the provision of appropriate nutritional options, including the role of calcium, carbohydrates and vitamin D in bone health is also important [50, 80]. The transitional time during puberty is an important period for primary prevention strategies. These strategies should focus on communication promoting variations in body shape, positive behaviours, and natural biological and psychological adaptations, with a de-emphasis on body weight and leanness [50].

Cumulative exposure to mechanical load (stress and strain) is the necessary cause for BSIs, and evidence suggests that rapid, large increases in training load can increase risk of BSIs [75, 81, 86]. Prevention strategies to mitigate this risk include well-structured progressive increases in training exposure, with less ‘spikes’ in training exposure [81], along with appropriate training exposure management and time between sessions to allow for the appropriate recovery of microdamage to bone [87]. An example of a universal prevention strategy in cricket, a sport with a high incidence of lumbar spine BSIs, is management of the number of balls athletes bowl each session [17, 81]. In some sports, managing training exposure is particularly pertinent leading up to camp-based situations, where athletes often perform training sessions consisting of increased frequency, volume and/or intensity compared with their usual training environment, where the workload applied may exceed an individual’s capacity to adapt [88].

To mitigate injury risk, training programs should focus on two key components: tissue-specific strength and tissue-specific stress and strain [86]. Evidence suggests that building soft tissue load capacity longer-term through the implementation of a resistance and plyometric program decreases the risk of BSIs through positive impact on bone biomarkers [27, 89]. If an athlete is exposed to congested periods of training with insufficient preparation, and without the appropriate development of soft tissue capacity, the risk for BSIs may increase substantially. However, how this sudden increase in training load relates to the development of BSIs is yet to be established [81].

### Biological Onset

The exact pathophysiology of BSIs is not conclusive, hence the development of prevention and intervention strategies currently relies on theoretical models [7, 90]. Figure 2 outlines one theory, where bones are exposed to cumulative mechanical load, resulting in bone strain [90]. Increased strain leads to increased accumulation of microdamage, thought to be the beginning of the pathology continuum [90]. With increased stress on the bone, it begins to deform within its elastic range, however it has the ability to return to its original configuration through targeted remodelling [90, 91]. Stress exceeding the elastic range creates plastic deformity and microfractures, leading to discontinuity within the cortical bone (stress fracture) [91]. Stress fractures can also occur as a result of cyclic overloading where there is insufficient time for remodelling to repair the damage caused, and where additional loading cycles enable further damage to accumulate [90]. A growing body of evidence suggests alignment between common engineering principles relating to mechanical fatigue and tissue response. However, it is important to acknowledge the contribution of physiological mechanisms in this process, including nutritional and hormonal, and these warrant future exploration [79, 92].

Bone has many functions, including the maintenance of mineral homeostasis and haematopoiesis [7]. Bone consists of two layers, an outer layer of hard, dense cortical bone and an inner core of trabecular bone [7]. Bone responds to stimuli through a continual remodelling process of resorption, through osteoclasts (breakdown bone), and formation through osteoblast activity, with this cycle occurring over a period of months [93]. Exposure to casual factors in the induction period results in a gradual imbalance to the bone remodelling and resorption cycle, until a threshold is met for biological onset of a BSI. Each of the component causes discussed have different induction periods, however, completion of a sufficient cause is synonymous with biological onset of a BSI [77]. Biological onset is the point at which the cumulative load exceeds the critical damage threshold for bones. The point of biological onset is difficult to determine, as symptoms are not yet apparent and the condition may remain undetected. Screening for the condition is used to identify signs that determine if biological onset has occurred. For example, is an individual in the susceptible stage, or do they have manifestation of the condition. The period from when biological onset occurs to when symptoms are first present is the latent period, which will be discussed in the sub-clinical phase.

### Sub-clinical Phase

The sub-clinical phase starts when mechanical load exceeds the critical damage threshold for bone, where fatigue failure occurs (biological onset), through to clinical manifestation of the condition. For BSIs this stage is where changes to the bone through an imbalance of targeted remodelling are occurring, where microdamage to the bone is greater than the rate of remodelling but the individual is asymptomatic. In the sub-clinical phase the condition is detectable with screening tests that identify signs of the disease, which determines if biological onset has occurred in the absence of disease symptoms [36]. Screening is a process of determining those who may have the condition in the population at risk. Those identified through screening require undergoing a diagnostic process to determine whether or not they have the condition (diagnosis). A screening test in this context is being applied at a population level, for example in breast cancer, where a defined population is at risk (women over a certain age). A screening program that would be introduced for BSIs would be applied to the entire population at risk (e.g. cricket fast bowlers). Judicious selection of a screening test is required and criteria such as the Wilson and Jungner criteria [94] may assist in this selection. This means the screening test must have the diagnostic statistics that support its use (that it can detect those with possible BSIs), it is safe to administer, is acceptable to the population being tested, and is applied within the latent period [94]. While there is an absence of evidence in BSIs it is recommended that these tests not be administered too close to exposure to suspected causal factors (e.g. a fast bowler in week one of the pre-season) as this may lead to a false negative on the screening test. It is important to note that a screening test would be applied to the population at risk and individuals with a positive test would then be followed up with individual care to rule in or out possible BSIs using diagnostic tests.

#### Clinical Manifestations (Signs, Detection Methods and Symptoms)

The progressive gradual onset of most BSIs and lack of an accepted screening tool presents challenges for early detection, hence clinical management and intervention of BSIs should be proactively aligned with the bone remodelling process. Pain reproduced during the physical examination is an important clinical feature of BSIs, but may not be diagnostic. However, variability in specific pain presentations exist between individuals [7, 71]. In selected sites (tibia, tarsals and metatarsals), tenderness associated with BSI is readily palpable, and on occasion, localised swelling and warmth may be evident [7]. In other sites, such as the femoral neck or the femoral shaft, symptoms are often more diffuse and complex, and clinical suspicion may be supported by symptom reproduction with certain bone loading physical examination tests including: hopping for lower extremity BSIs [95], the fulcrum test for femoral diaphysis or shaft BSIs [96] or the squeeze test for calcaneal BSIs [97]. In tibial BSIs, hopping on the affected leg is reported to be 100% sensitive in ruling in a BSI in military recruits; however, this test has low specificity, meaning a large number of false positive tests occur [98].

The use of magnetic resonance imaging (MRI) can assist in confirming the diagnosis and grading the severity of the injury, which may be useful to inform injury management. Bone oedema can be visualised on MRI, and typically presents in the fat-supressed (T2-weighted) images as diffuse, irregular and hyperintense signal, in conjunction with a corresponding hypointense signal on the T1-weighted images [7]. In advanced BSI cases, the presence of a radiological fracture line is visible [7]. Numerous radiographic classification systems exist to assess BSI severity. However, the Fredericson et al. [99] system for tibial BSIs is the most widely adopted classification system, which describes a continuum of injury from low to high grade on the basis of specific MRI criteria.

Athletes with BSIs typically present with gradual onset activity-related pain, consistent with overuse injuries, with symptoms varying depending where on the BSI continuum an individual presents [71, 80]. Depending on the stage of the injury, pain ranges in severity, often starting as a diffuse mild ache after activity, through to a localised sharp pain reproduced each time the affected bone is loaded [7, 71]. Early in the injury continuum BSIs are often pain-free when unloaded, and thus, these injuries are commonly ignored initially [80]. However, with continued loading and pathology progression, pain often intensifies in severity and presents at an earlier stage during activity [71, 80]. With further BSI progression, pain may be present for longer periods after activity has ceased, and may then start to be present with activities associated with lower levels of bone loading, including walking [80]. Ultimately, pain will impact on an individual’s ability to participate in any physical activity, with the potential requirement to cease training [90]. In the advanced stages, night pain and pain at rest can be present [7, 71, 80].

#### Secondary Prevention

Secondary prevention focuses on early detection and early intervention, moving the time to usual diagnosis and treatment forward, with the aim to prevent the progression and worsening of the condition [2, 81]. Secondary prevention involves screening for sub-clinical signs or markers, which facilitates delivery of appropriate and available early intervention aimed to reduce the severity of the condition [100]. Secondary prevention of BSIs first involves identifying subgroups of athletes at higher risk of sub-clinical disease/injury. These athletes can then undergo sensitive screening tests if clinically indicated [101]. Individuals or subgroups who have higher probabilities to be at the stage where the biological onset for bone has been exceeded should be prioritised for screening.

To implement effective secondary prevention programs, appropriate and timely screening tests are needed to identify and detect the condition before usual diagnosis. Figure 2a is a theoretical concept of the clinical course of BSIs utilising the natural history of disease framework, where standard clinical care is applied. The natural history and clinical course differ when an intervention is applied by health and medical personnel to decrease the duration of BSIs. Figure 2b outlines a proposed early intervention model for BSIs when a screening test is applied within the sub-clinical phase of the natural history in addition to subsequent treatment which would otherwise occur following later diagnosis. The application of an appropriate and effective screening measures leads to early diagnosis and subsequent early intervention to reduce the clinical course [102]. Screening for BSIs would help to determine whether an individual is in the stress reaction phase, where they may be asymptomatic or symptomatic, or may detect further progression along the pathology continuum and identify a stress fracture [90]. Magnetic resonance imaging is used in some sports, such as cricket, to screen for bone marrow oedema in asymptomatic junior cricket fast bowlers [17]. Bone marrow oedema detected on MRI may precede the progression from presymptomatic bone stress to symptomatic stress fractures, with bone marrow oedema possibly present for months prior to symptoms appearing [17, 43]. Junior cricketers with bone marrow oedema detected on MRI have been reported to experience a 20 times greater risk of sustaining a lumbar spine stress fracture during the season compared with those athletes without bone marrow oedema [17]. However, there are limitations to this screening method, and using the unquantified presence of bone marrow oedema on MRI holds poor positive predictive value for progression to BSI in adult cricketers [103]. Measuring signal intensity on MRI is a proposed method to increase accuracy of quantifying bone marrow oedema as a potential screening test [103]. Future studies with larger cohorts and longitudinal designs are required for a better understanding of the association between screening bone marrow oedema intensity and the risk of developing a BSI across all age groups and sexes [103]. There is no current imaging modality that is able to directly quantify the amount of micro damage in bone [104]. Dual-energy x-ray absorptiometry (DEXA) is used to characterise bone health, however a DEXA scan is unable to assess the risk of BSIs owing to its inability to measure bone geometry or microarchitecture, thus there is no current recommendation to use DEXA on a routine basis as a screening tool [7].

A shin palpation test and shin oedema test have demonstrated strong predictive ability for the future onset of medial tibial stress syndrome (MTSS) symptoms [105]. Medial tibial stress syndrome can be a predecessor to tibial BSIs. When screening for MTSS in military recruits, a positive shin palpation test or a positive shin oedema test appears to be a strong predictor for an individual developing MTSS in the future, which may be applicable in athletic populations [105] and is currently untested. Shin palpation tests may provide a screening test option for superficial BSIs where the bone is easily palpable, but for regions where direct bone palpation is not possible (femoral neck and femoral shaft) these tests would not be appropriate.

Whilst the theoretical basis for secondary prevention for BSIs is established, future research should focus on identifying an appropriate screening test within the latent period of disease. For secondary prevention to occur, a screening test should only be applied if it leads to early intervention. This helps to inform whether screening for BSIs is worthwhile and feasible. To assess the appropriateness of screening for BSIs, the World Health Organisation Principles for screening developed by Wilson and Jungner [94], provide criteria that can be applied by practitioners to identify key issues that should be considered when assessing potential population based screening programs. To assess how this might be applied in the field, the paper by Kountouris et al. [43] has been used as a simple example (Table 1).

**Table 1:** Application of the World Health Organisation principles for screening

Criteria	Assessment: elite cricket [43]
The condition should be an important health problem	Yes, high burden injury for the population of interest
There should be an accepted treatment for patients with recognised disease	Yes
Facilities for diagnosis and treatment should be available	Yes
There should be recognisable latent and early symptomatic stage	Yes
There should be a suitable test or examination	Yes—serial MRI performed (six MRIs in 8 months)
The test should be acceptable to the population	Yes, serial MRIs were an acceptable test
The natural history of the condition including development from latent to declared disease should be adequately understood	BSIs are well researched and understood, however this information has not yet been applied to the natural history of disease
There should be an agreed upon policy on whom to treat as patients	Yes, this was decided on prior to screening
The cost of case-finding (including diagnosis and treatment of patients diagnosed) should be economically balanced in relation to possible expenditure on medical care as a whole	Yes, appropriate resources and funding were prioritised, as BSIs are a high burden injury in the population of interest
Case-finding should be a continuing process and not a ‘once and for all’ project	Yes, the screening program was implemented continually throughout the season

## Diagnosis

Diagnosis is a critical time point in the natural history, where the individual seeks care and is diagnosed with the disease by a health professional. Clinical suspicion of BSIs is achieved through a combination of a patient history and physical examination, with imaging used to confirm and grade the injury [71]. The features already outlined in Sect. 3.4.1 can assist clinicians with confirming a diagnosis.

### Tertiary Prevention

The construct of tertiary prevention, beginning following a diagnosis, encompasses treatment, management of known complications and promoting rehabilitation to reduce the impact of both short and long-term consequences. In the context of BSIs, tertiary interventions designed to prevent further health complications should be prioritised for injured athletes, given the high incidence of recurrence of BSIs [106]. Identification of likely casual factors for BSIs should occur so that treatment can be individualised and targeted to optimise bone healing and recovery, decrease subsequent injury risk and facilitate a safe return to sports participation [7]. 

Treatment for BSIs consists of three main phases: (1) effective bone remodelling, (2) progressive exposure to types of mechanical stressors through targeted exercise and progressive exposure to sports training in a structured manner and (3) a structured return to sport program, with particular attention to the absence of pain throughout each phase [7]. The management of BSIs is guided by the location of the injury, and can be classified as either low risk (e.g. second and third metatarsal shaft, posteromedial tibia, fibula, ribs and humeral shaft) or high risk (e.g. base of fifth metatarsal, sesamoid, talus, medial malleolus, navicular, anterior tibia, patella, femoral neck and lumbar spine) [7]. Careful monitoring of high risk BSIs must occur during rehabilitation because the potential for delayed union, non-healing, complete fracture or delayed returned to sport is high [107]. Initial management for BSIs includes a period of cessation from loading activity to the site to allow for bone healing and prevent progression of pathology [80]. During the rehabilitation phase, pain monitoring can be useful in determining the healing response and can guide specific safe activities at each phase of recovery [80, 108]. The extent and duration of activity modification is highly variable and decided on a case-by-case basis, but can range from weeks to months following diagnosis [80].

In this phase clinicians should explore the antecedent causes (causes of the causes) of the BSI, as outlined in Sect. 3.2.1, which led to the decrease in tissue strength and the exposure to cumulative mechanical load (cycles, frequency, magnitude), so these factors can be addressed. Owing to the multifactorial nature of BSIs, a multidisciplinary approach with a clinical team consisting of a doctor, dietitian, psychologist and physiotherapist should be considered for adequate treatment to occur. Where indicated, treatment approaches should address the cause of problematic LEA, which is the underlying cause of the female athlete triad [58]. Energy status is recommended to be restored and regulated through diet and training interventions, with a focus on normalisation of body mass to enable successful resumption of an individual’s menstrual cycle, which leads to improved bone health [58, 109, 110]. Energy availability factors and oestrogen deficiency are the underlying aetiology of bone loss in females with menstrual disturbance, hence increasing body mass and subsequent resumption of menses is critical to prevent further reduction in bone mineral density [111–113]. There are significant improvements in bone health outcomes in case studies of amenorrhoeic female athletes who were able to increase their body mass [114, 115]. If a cause of LEA is inadvertent undereating, then referral to a sports dietitian for nutritional education is appropriate [59]. If a cause for LEA is disordered eating behaviour, involving a physician is recommended, in conjunction with a sports dietitian for nutritional counselling and education [59]. If a cause for LEA involves a clinical eating disorder or compulsive exercise behaviours, treatment should consist of assessment and management with a physician, nutritional counselling and education with a sports dietitian and psychologist referral for appropriate psychological treatment [58, 71, 116].

Graduated return approaches to high mechanical loading activities are introduced in the initial phase of rehabilitation with the progressive return to activity dependent on the absence of symptoms during daily activities [80, 108]. Whilst there are several published return to sport protocols [80, 117], there is no established return to high mechanical loading activities for BSIs. Current recommendations suggest the optimal strategy for return to high mechanical loading activities should centre around collaborative decision making on the basis of the individual athlete [118].

## Integrated Strategy of Prevention and Treatment

This paper has proposed a structured integrated prevention model to direct which individuals receive specific interventions in what phase of the natural history for BSIs. This model allows sports professionals to work in a collaborative manner to design primary prevention programs focused on risk and protective factors, which are related to sports specific factors (exposure). Secondary prevention programs are implemented primarily to reduce the burden of BSIs in a population at risk based on their exposure. To determine which individuals are considered at risk, a two-phase approach can be implemented: (1) identify those individuals who require intervention and (2) follow a process that leads to an earlier diagnosis than what would occur naturally, through a deliberate screening program to screen at risk individuals. Once diagnosed, the tertiary prevention phase allows sports professionals to work in a multidisciplinary team to prevent further health complications and address the antecedent causes of BSIs at an individual level.

## Conclusions

Despite the amount of evidence on BSIs, and previous efforts to synthesise this body of work, it has not yet been integrated and applied to the natural history of disease. This paper combines models of prevention to provide clinicians working with athletic populations a framework to utilise, unifying the current evidence into tangible actions for evidence-based prevention. This construct identifies opportunities to optimise outcomes for BSIs; however, it requires empirical validation before it is implemented at scale.

## References

[1] Wentz L, Liu P, Haymes E, Ilich JZ. Females have a greater incidence of stress fractures than males in both military and athletic populations: a systemic review. Mil Med. 2011;176(4):420–30. 21539165 10.7205/milmed-d-10-00322

[2] Jacobsson J, Timpka T. Classification of prevention in sports medicine and epidemiology. Sports Med. 2015;45(11):1483–7. 26245875 10.1007/s40279-015-0368-x

[3] Ruddick GK, Lovell GA, Drew MK, Fallon KE. Epidemiology of bone stress injuries in Australian high performance athletes: a retrospective cohort study. J Sci Med Sport. 2019;22(10):1114–8. 31307905 10.1016/j.jsams.2019.06.008

[4] Trease L, Wilkie K, Lovell G, Drew M, Hooper I. Epidemiology of injury and illness in 153 Australian international-level rowers over eight international seasons. Br J Sports Med. 2020;54:1288–93. 32586943 10.1136/bjsports-2019-101402

[5] Changstrom BG, Brou L, Khodaee M, Braund C, Dawn CR. Epidemiology of stress fracture injuries among US high school athletes, 2005–2006 through 2012–2013. Am J Sports Med. 2015;43(1):26–33. 25480834 10.1177/0363546514562739

[6] Rizzone KH, Ackerman KE, Roos KG, Dompier TP, Kerr ZY. The epidemiology of stress fractures in collegiate student-athletes, 2004–2005 through 2013–2014 academic years. J Athl Train. 2017;52:966–75. 28937802 10.4085/1062-6050-52.8.01 PMC5687241

[7] Hoenig T, Ackerman KE, Beck BR, Bouxsein ML, Burr DB, Hollander K, et al. Bone stress injuries. Nat Rev. 2022;8(26):1–20. 10.1038/s41572-022-00352-y PMC13227457 35484131

[8] Kelly S, Waring A, Stone B, Pollock N. Epidemiology of bone injuries in elite athletics: a prospective 9-year cohort study. Phys Ther Sport. 2024;66:67–75. 38340615 10.1016/j.ptsp.2024.01.005

[9] Eales B, Jones N, Saw A, Obst A, Smith M, Kountouris A, et al. Lumbar bone stress injuries in elite Australian cricket players: a comprehensive case series. J Sci Med Sport. 2022;25:S79–80. 10.1097/JSM.0000000000001132 36853903

[10] Wright AA, Taylor JB, Ford KR, Siska L, Smoliga JM. Risk factors associated with lower extremity stress fractures in runners: a systematic review with meta-analysis. Br J Sports Med. 2015;49(23):1517–23. 26582192 10.1136/bjsports-2015-094828

[11] Asano L, Duarte A, Silva A. Stress fractures in the foot and ankle of athletes. Rev Assoc Méd Bras. 2014;60(6):512–7. 25650848 10.1590/1806-9282.60.06.006

[12] Caesar BC, McCollum GA, Elliot R, Williams A, Calder JDF. Stress fractures of the tibia and medial malleolus. Foot Ankle Clin. 2013;18(2):339–55. 23707181 10.1016/j.fcl.2013.02.010

[13] Joy EA, Campbell D. Stress fractures in the female athlete. Curr Sports Med Rep. 2005;4(6):323–8. 16282034 10.1097/01.csmr.0000306294.72578.a8

[14] Hoenig T, Eissele J, Strahl A, Popp KL, Stürznickel J, Ackerman KE, et al. Return to sport following low-risk and high-risk bone stress injuries: a systematic review and meta-analysis. Br J Sports Med. 2023;0:1–8. 10.1136/bjsports-2022-106328 36720584

[15] Crunkhorn ML, Toohey LA, Charlton P, Drew M, Watson K, Etxebarria N. Injury incidence and prevalence in elite short-course triathletes: a 4-year prospective study. Br J Sports Med. 2024;58:470–6. 38331566 10.1136/bjsports-2023-107327

[16] Guevara SA, Crunkhorn ML, Drew M, Waddington G, Périard JD, Etxebarria N, et al. Injury and illness in short-course triathletes: a systematic review. J Sport Health Sci. 2023;13(2):172–85. 36898525 10.1016/j.jshs.2023.03.002 PMC10980869

[17] Kountouris A, Sims K, Beakley D, Rotstein A, Orchard J, Cook J. Bone marrow oedema on MRI is associated with increased risk of lumbar stress fracture in junior cricket fast bowlers. J Sci Med Sport. 2017;20S:40–2.

[18] Myrick KM, Myrick SA, Ezomo O. Ulnar stress fracture in a softball player. Clin Case Rep. 2020;8:1547–52. 32884793 10.1002/ccr3.2933 PMC7455444

[19] Silva RT, Hartmann LG, de Souza Laurino CF. Stress reaction of the humerus in tennis players. Br J Sports Med. 2007;41:824–6. 17957022 10.1136/bjsm.2006.034942 PMC2465260

[20] Hollander K, Rahlf AL, Wilke J, Edler C, Steib S, Junge A, et al. Sex-specific differences in running injuries: a systematic review with meta-analysis and meta-regression. Sports Med. 2021;51:1011–39. 33433864 10.1007/s40279-020-01412-7 PMC8053184

[21] Rauh MJ, Barrack M, Nichols JF. Associations between the female athlete triad and injury among high school runners. Int J Sports Phys Ther. 2014;9:948–58. 25540710 PMC4275199

[22] Tenforde AS, Carlson JL, Chang A, Sainani KL, Shultz R, Kim JH, et al. Association of the female athlete triad risk assessment stratification to the development of bone stress injuries in collegiate athletes. Sports Med. 2017;45:302–10. 10.1177/0363546516676262 28038316

[23] Ackerman KE, Cano Sokoloff N, De Nardo MG, Clarke HM, Lee H, Misra M. Fractures in relation to menstrual status and bone parameters in young athletes. Med Sci Sports Exer. 2015;47(8):1577–86. 10.1249/MSS.0000000000000574 PMC4430468 25397605

[24] Prather H, Hunt D, McKeon K, Simpson S, Meyer EB, Yemm T, et al. Are elite female soccer athletes at risk for disordered eating attitudes, menstrual dysfunction, and stress fractures? PM R. 2016;8(3):208–13. 26188245 10.1016/j.pmrj.2015.07.003 PMC8301748

[25] Mountjoy M, Sundgot-Borgen J, Burke L, Carter S, Constantini N, Lebrun C, et al. The IOC consensus statement: beyond the Female Athlete Triad—relative energy deficiency in sport (RED-S). Br J Sports. 2014;48:491–7. 10.1136/bjsports-2014-093502 24620037

[26] Nose-Ogura S, Yoshino O, Dohi M, Kigawa M, Harada M, Hiraike O, et al. Risk factors of stress fractures due to the female athlete triad: differences in teens and twenties. Scand J Med Sci Sports. 2019;29(10):1501–10. 31100189 10.1111/sms.13464

[27] Tenforde AS, Fredericson M, Sayres LC, Cutti P, Sainani KL. Identifying sex-specific risk factors for low bone mineral density in adolescent runners. Am J Sports Med. 2015;43(6):1494–504. 25748470 10.1177/0363546515572142

[28] De Souza MJ, Williams NI, Nattiv A, Joy E, Misra M, Loucks AB, et al. Misunderstanding the Female Athlete Triad: refuting the IOC Consensus Statement on Relative Energy Deficiency in Sport (RED-S). Br J Sports Med. 2014;48(20):1461–5. 25037200 10.1136/bjsports-2014-093958

[29] Welck MJ, Hayes T, Pastides P, Khan W, Rudge B. Stress fractures of the foot and ankle. Injury. 2017;48(8):1722–6. 26412591 10.1016/j.injury.2015.06.015

[30] Barrack MT, Fredericson M, Tenforde AS, Nattiv A. Evidence of a cumulative effect for risk factors predicting low bone mass among male adolescent athletes. Br J Sports Med. 2017;51:200–5. 29461218 10.1136/bjsports-2016-096698

[31] Barrack MT, Gibbs JC, De Souza MJ, Williams NI, Nichols JF, Rauth MJ, et al. Higher incidence of bone stress injuries with increasing female athlete triad-related risk factors: a prospective multisite study of exercising girls and women. Am J Sports Med. 2014;42(4):949–58. 24567250 10.1177/0363546513520295

[32] Field AE, Gordon CM, Pierce LM, Ramappa A, Kocher MS. Prospective study of physical activity and risk of developing a stress fracture among preadolescent and adolescent girls. Arch Pediatr Adolesc Med. 2011;165:723–8. 21464375 10.1001/archpediatrics.2011.34 PMC3132304

[33] Kenneally M, Casado A, Gomez-Ezeiza J, Santos-Concejero J. Training characteristics of a World Championship 5000-m finalist and multiple continental record holder over the year leading to a World Championship final. Sports Physiol Perform. 2021;17(1):142–6. 10.1123/ijspp.2021-0114 34426556

[34] Tjelta LI, Enoksen E. Training characteristics of male junior cross country and track runners on European top level. Int J Sports Sci Coach. 2010;5(2):193–203.

[35] Last JM. Dictionary of epidemiology. New York: Oxford University Press; 1988.

[36] Porta M. A Dictionary of epidemiology. 6th ed. Oxford: Oxford University Press; 2014.

[37] Centers for Disease Control and Prevention. Principles of epidemiology. 2nd ed. Atlanta: U.S. Department of Health and Human Services; 1992.

[38] Burns J, Keenan A-M, Redmond AC. Factors associated with triathlon-related overuse injuries. J Orthop Sports Phys Ther. 2003;33(4):177–84. 12723674 10.2519/jospt.2003.33.4.177

[39] McHardy A, Pollard H, Fernandez M. Triathlon injuries: a review of the literature and discussion of potential injury mechanisms. Clin Chiropr. 2006;9(3):129–38.

[40] Vleck VE, Bentley DJ, Millet GP, Cochrane T. Triathlon event distance specialization: training and injury effects. J Strength Cond Res. 2010;24(1):30–6. 20042924 10.1519/JSC.0b013e3181bd4cc8

[41] Windt J, Gabbett TJ. How do training and competition workloads relate to injury? The workload—injury aetiology model. Br J Sports Med. 2017;51:428–35. 27418321 10.1136/bjsports-2016-096040

[42] Olivier B, Taljaard T, Burger E, Brukner P, Orchard J, Gray J, et al. Which extrinsic and intrinsic factors are associated with non-contact injuries in adult cricket fast bowlers? Sports Med. 2016;46:79–101. 26365510 10.1007/s40279-015-0383-y

[43] Kountouris A, Sims K, Beakley D, Saw AE, Orchard J, Rotstein A, et al. MRI bone marrow oedema precedes lumbar bone stress injury diagnosis in junior elite cricket fast bowlers. Br J Sports Med. 2019;53:1236–9. 30425044 10.1136/bjsports-2017-097930

[44] Meeuwisse WH, Tyreman H, Hagel B, Emery C. A dynamic model of etiology in sport injury: the recursive nature of risk and causation. Clin J Sport Med. 2007;17:215–9. 17513916 10.1097/JSM.0b013e3180592a48

[45] Bennell KL, Malcolm SA, Thomas SA, Wark JD, Brukner PD. The incidence and distribution of stress fractures in competitive track and field athletes. A twelve-month prospective study. Am J Sports Med. 1996;24:211–7. 8775123 10.1177/036354659602400217

[46] Pepper M, Akuthota V, McCarty EC. The pathophysiology of stress fractures. Clin Sports Med. 2006;25(1):1–16. 16324969 10.1016/j.csm.2005.08.010

[47] DiFiori JP, Benjamin HJ, Brenner JS, Gregory A, Jayanthi N, Landry GL, et al. Overuse injuries and burnout in youthsports: a position statement from the American Medical Society for Sports Medicine. Br J Sports Med. 2014;48:287–8. 24463910 10.1136/bjsports-2013-093299

[48] Cipriani DJ, Swartz JD, Hodgson CM. Triathlon and the multisport athlete. J Orthop Sports Phys Ther. 1998;27(1):42–50. 9440040 10.2519/jospt.1998.27.1.42

[49] Duckham RL, Brooke-Wavell K, Summers GD, Cameron N, Peirce N. Stress fracture injury in female endurance athletes in the United Kingdom: a 12-month prospective study: stress fractures in female endurance athletes. Scand J Med Sci Sports. 2015;25:854–9. 25892560 10.1111/sms.12453

[50] Mountjoy M, Ackerman KE, Bailey DM, Burke LM, Constantini N, Hackney AC, et al. 2023 International Olympic Committee’s (IOC) consensus statement on Relative Energy Deficiency in Sport (REDs). Br J Sports Med. 2023;57:1073–97. 37752011 10.1136/bjsports-2023-106994

[51] Edouard P, Feddermann-Demont N, Alonso JM, Branco P, Junge A. Sex differences in injury during top-level international athletics championships: surveillance data from 14 championships between 2007 and 2014. Br J Sports Med. 2015;49(7):472–7. 25618889 10.1136/bjsports-2014-094316

[52] Marx JJM. Iron deficiency in developed countries: prevalance, influence of lifestyle factors and hazards of prevention. Eur J Clin Nutr. 1997;51:491–4. 11248872 10.1038/sj.ejcn.1600440

[53] He C-S, Handzlik M, Fraser WD, Muhamad A, Preston H, Richardson A, et al. Influence of vitamin D status on respiratory infection incidence and immune function during 4 months of winter training in endurance sport athletes. Exerc Immunol Rev. 2013;19:86–101. 23977722

[54] Shuler FD, Wingate MK, Moore GH, Giangarra C. Sports health benefits of vitamin D. Sports Health. 2012;4:496–501. 24179588 10.1177/1941738112461621 PMC3497950

[55] Tenforde AS, Sayres LC, McCurdy ML, Sainani KL, Fredericson M. Identifying sex-specific risk factors for stress fractures in adolescent runners. Med Sci Sports Exer. 2013;45(10):1843–51. 10.1249/MSS.0b013e3182963d75 23584402

[56] Nattiv A, Kennedy G, Barrack MT, Abdelkerim A, Goolsby MA, Arends JC, et al. Correlation of MRI grading of bone stress injuries with clinical risk factors and return to play: a 5-year prospective study in collegiate track and field athletes. Am J Sports Med. 2013;41(8):1930–41. 23825184 10.1177/0363546513490645 PMC4367232

[57] Drinkwater BL, Nilson K, Ott S, Chesnut CH. Bone mineral density after resumption of menses in amenorrheic athletes. JAMA. 1986;256:380–2. 3723725

[58] Nattiv A, Loucks AB, Manore MM, Sanborn CF, Sundgot-Borgen J, Warren MP. American College of Sports Medicine position stand. The female athlete triad. Med Sci Sports Exerc. 2007;39:1867–82. 17909417 10.1249/mss.0b013e318149f111

[59] De Souza MJ, Nattiv A, Joy E, Misra M, Williams NI, Mallinson RJ, et al. 2014 Female Athlete Triad Coalition Consensus Statement on Treatment and Return to Play of the Female Athlete Triad: 1st international conference held in San Francisco, California, May 2012 and 2nd international conference held in Indianapolis, Indiana, May 2013. Br J Sports Med. 2014;48:289–309. 24463911 10.1136/bjsports-2013-093218

[60] Duckham RL, Peirce N, Meyer C, Summers GD, Cameron N, Brooke-Wavell K. Risk factors for stress fracture in female endurance athletes: a cross-sectional study. BMJ Open. 2012;2(6):1–7. 10.1136/bmjopen-2012-001920 PMC3533057 23166136

[61] Kelsey JL, Bachrach LK, Procter-Gray E, Nieves J, Greendale GA, Sowers M, et al. Risk factors for stress fracture among young female cross-country runners. Med Sci Sports Exerc. 2007;39:1457–63. 17805074 10.1249/mss.0b013e318074e54b

[62] Lauder TD, Dixit S, Pezzin LE, Williams MV, Campbell CS, Davis GD. The relation between stress fractures and bone mineral density: evidence from active-duty army women. Arch Phys Med Rehabil. 2000;81:73–9. 10638880 10.1016/s0003-9993(00)90225-9

[63] Rauh MJ, Macera CA, Trone DW, Shaffer RA, Brodine SK. Epidemiology of stress fracture and lower-extremity overuse injury in female recruits. Med Sci Sports Exerc. 2006;38:1571–7. 16960517 10.1249/01.mss.0000227543.51293.9d

[64] Williams NI, Helmreich DL, Parfitt DB, Caston-Balderrama A, Cameron JL. Evidence for a causal role of low energy availability in the induction of menstrual cycle disturbances during strenuous exercise training. J Clin Endocrinol Metab. 2001;86:5184–93. 11701675 10.1210/jcem.86.11.8024

[65] Tomten SE, Falch JA, Birkeland KI, Hemmersbach P, Høstmark AT. Bone mineral density and menstrual irregularities. A comparative study on cortical and trabecular bone structures in runners with alleged normal eating behavior. Int J Sports Med. 1998;19(2):92–7. 9562216 10.1055/s-2007-971888

[66] Kuh D, Ben-Shlomo Y, Lynch J, Hallqvist J, Power C. Life course epidemiology. J Epidemiol Community Health. 2003;57:778–83. 14573579 10.1136/jech.57.10.778 PMC1732305

[67] Weaver CM. The role of nutrition on optimizing peak bone mass. Asia Pac J Clin Nutr. 2008;17:135–7. 18296321

[68] Myer GD, Jayanthi N, DiFiori JP, Faigenbaum AD, Kiefer AW, Logerstedt D, et al. Sports specialization, part II: alternative solutions to early sport specialization in youth athletes. Sports Health. 2016;8:65–73. 26517937 10.1177/1941738115614811 PMC4702158

[69] Fredericson M, Ngo J, Cobb K. Effects of ball sports on future risk of stress fracture in runners. Clin J Sport Med. 2005;15:136–41. 15867555 10.1097/01.jsm.0000165489.68997.60

[70] Tenforde AS, Sainani KL, Carter Sayres L, Milgrom C, Fredericson M. Participation in ball sports may represent a prehabilitation strategy to prevent future stress fractures and promote bone health in young athletes. PM&R. 2015;7:222–5. 25499072 10.1016/j.pmrj.2014.09.017

[71] Beck B, Drysdale L. Risk factors, diagnosis and management of bone stress injuries in adolescent athletes: a narrative review. Sports. 2021;9(4):52–76. 33923520 10.3390/sports9040052 PMC8073721

[72] Hulme A, Finch CF. From monocausaility to systems thinking: a complementary and alternative conceptual approach for better understanding the development and prevention of sports injury. Inj Epidemiol. 2015;2:31–43. 26691678 10.1186/s40621-015-0064-1 PMC4673096

[73] Bittencourt NFN, Meeuwisse WH, Mendonça LD, Nettel-Aguirre A, Ocarino JM, Fonseca ST. Complex systems approach for sports injuries: moving from risk factor identification to injury pattern recognition narrative review and new concept. Br J Sports Med. 2016;50:1309–14. 27445362 10.1136/bjsports-2015-095850

[74] Kalkhoven J. Athletic injury research: frameworks, models and the need for casual knowledge. Sports Med. 2024;54:1121–37. 38507193 10.1007/s40279-024-02008-1 PMC11127898

[75] Kalkhoven JT, Watsford ML, Coutts AJ, Edwards WB, Impellizzeri FM. Training load and injury: causal pathways and future directions. Sports Med. 2021;51:1137–50. 33400216 10.1007/s40279-020-01413-6

[76] Wensink M, Westendorp RGJ, Baudisch A. The causal pie model: an epidemiological method applied to evolutionary biology and ecology. Ecol Evol. 2014;4(10):1924–30. 24963386 10.1002/ece3.1074 PMC4063485

[77] Rothman KJ. Causes. Am J Epidemiol. 1976;104:587–92. 998606 10.1093/oxfordjournals.aje.a112335

[78] Wolff J. The law of bone remodeling. Berlin: Springer; 1986. (**translation of the German 1892 edition**).

[79] Gallagher S, Schall MCJ. Musculoskeletal disorders as a fatigue failure process: evidence, implications and research needs. Ergonomics. 2017;60(2):255–69. 27376409 10.1080/00140139.2016.1208848

[80] Warden SJ, Davis IS, Fredericson M. Management and prevention of bone stress injuries in long-distance runners. J Orthop Sports Phys Ther. 2014;44(10):749–65. 25103133 10.2519/jospt.2014.5334

[81] Drew M, Cook J, Finch C. Sports-related workload and injury risk: simply knowing the risks will not prevent injuries: narrative review. Br J Sports Med. 2016;50:1306–8. 27166288 10.1136/bjsports-2015-095871

[82] Torstveit MK, Ackerman KE, Constantini N, Holtzman B, Koehler K, Mountjoy ML, et al. Primary, secondary and tertiary prevention of Relative Energy Deficiency in Sport (REDs): a narrative review by a subgroup of the IOC consensus on REDs. Br J Sports Med. 2023;57:1119–26. 37752004 10.1136/bjsports-2023-106932

[83] Gillbanks L, Mountjoy M, Filbay SR. Insufficient knowledge and inapproriate physiotherapy management of relative energy deficiency in sport (RED-S) in lightweight rowers. Phys Ther Sport. 2022;54:8–15. 34929534 10.1016/j.ptsp.2021.12.002

[84] Fahrenholtz IL, Melin AK, Garthe I, Hollekim-Strand SM, Ivarsson A, Koehler K, et al. Effects of a 16-week digital intervention on sports nutrition knowledge and behavior in female endurance athletes with risk of relative energy deficiency in sport (REDs). Nutrients. 2023;15:1082–2002. 36904082 10.3390/nu15051082 PMC10005555

[85] Stewart TM, Pollard T, Hildebrandt T, Wesley NY, Kilpela LS, Becker CB. The female athlete body project study: 18-month outcomes in eating disorder symptoms and risk factors. Int J Eat Disord. 2019;52:1291–300. 31350934 10.1002/eat.23145 PMC7089677

[86] Impellizzeri FM, Menaspa P, Coutts AJ, Kalkhoven J, Menaspa MJ. Training load and its role in injury prevention, part I: back to the future. J Athl Train. 2020;55(9):885–92. 32991701 10.4085/1062-6050-500-19 PMC7534945

[87] Lennox GM, Wood PM, Schram B, Canetti EFD, Simas V, Pope R, et al. Non-modifiable risk factors for stress fractures in military personnel undergoing training: a systematic review. Int J Environ Res Public Health. 2022;19:422–43. 10.3390/ijerph19010422 PMC8744653 35010681

[88] Gabbett T. The training-performance puzzle: how can the past inform future training directions? J Athl Train. 2020;55(9):874–84. 32991700 10.4085/1062/6050.422.19 PMC7534940

[89] Nattiv A. Stress fractures and bone health in track and field athletes. J Sci Med Sport. 2000;3:268–79. 11101266 10.1016/s1440-2440(00)80036-5

[90] Warden SJ, Burr DB, Burkner PD. Stress fractures: pathophysiology, epidemiology, and risk factors. Curr Osteoporos Rep. 2006;4:103–9. 16907999 10.1007/s11914-996-0029-y

[91] Matcuk GR, Mahanty SR, Skalski MR, Patel DB, White EA, Gottsegen CJ. Stress fractures: pathophysiology, clinical presentation, imaging features, and treatment options. Emerg Radiol. 2016;23(4):365–75. 27002328 10.1007/s10140-016-1390-5

[92] Gallagher S, Heberger JR. Examining the interaction of force and repetition on musculoskeletal disorder risk: a systematic literature review. Hum Factors. 2013;55(1):108–24. 23516797 10.1177/0018720812449648 PMC4495348

[93] Dolan E, Varley I, Ackerman KE, Pereira RMR, Elliott-Sale KJ, Sale C. The bone metabolic response to exercise and nutrition. Exerc Sport Sci Rev. 2020;48(2):49–58. 31913188 10.1249/JES.0000000000000215

[94] Wilson JMG, Jungner G. Principles and practice of screening for disease. Geneva: WHO; 1968.

[95] Matheson GO, Clement DB, McKenzie DC, Taunton JE, Lloyd-Smith DR, MacIntyre JG. Stress fractures in athletes. A study of 320 cases. Am J Sports Med. 1987;15:46–58. 3812860 10.1177/036354658701500107

[96] Johnson AW, Weiss CB, Wheeler DL. Stress fractures of the femoral shaft in athletes—more common than expected: a new clinical test. Am J Sports Med. 1994;22:248–56. 8198195 10.1177/036354659402200216

[97] Madden C, Mellion M. Sever’s disease and other causes of heel pain in adolescents. Am Fam Phys. 1996;54:1995–2000. 8900359

[98] Milgrom C, Zloczower E, Fleischmann C, Spitzer E, Landau R, Bader T, et al. Medial tibial stress fracture diagnosis and treatment guidelines. J Sci Med Sport. 2021;24:526–30. 33298373 10.1016/j.jsams.2020.11.015

[99] Fredericson M, Bergman AG, Hoffman KL, Dillingham MS. Tibial stress reaction in runners. Correlation of clinical symptoms and scintigraphy with a new magnetic resonance imaging grading system. Am J Sports Med. 1995;23:472–81. 7573660 10.1177/036354659502300418

[100] Giesecke J. Modern infectious disease epidemiology. London: Taylor and Francis Ltd; 2001.

[101] Chen YT, Tenforde AS, Fredericson M. Update on stress fractures in female athletes: epidemiology, treatment, and prevention. Curr Rev Musculoskelet Med. 2013;6(2):173–81. 23536179 10.1007/s12178-013-9167-x PMC3702771

[102] Gordis L. Epidemiology. 5th ed. Philadelphia: Saunders/Elsevier; 2014.

[103] Bell AJ, Nunnerley JL, Shackel DF, Coates MH, Campbell RG, Frampton CM, et al. Is MRI screening for bone marrow oedema useful in predicting lumbar bone stress injuries in adultmale professional cricketers? A NewZealand pilot study. J Sci Med Sport. 2023;26:410–4. 37541867 10.1016/j.jsams.2023.06.013

[104] Seref- Ferlengez Z, Kennedy OD, Schaffler MB. Bone microdamage, remodeling and bone fragility: how much damage is too much damage? Bone key Rep. 2015;4:644. 10.1038/bonekey.2015.11 PMC4371415 25848533

[105] Newman P, Adams R, Waddington G. Two simple clinical tests for predicting onset of medial tibial stress syndrome: shin palpation test and shin oedema test. Br J Sports Med. 2012;46:861–4. 22966153 10.1136/bjsports-2011-090409

[106] Nabhan D, Taylor D, Lewis M, Bahr R. Protecting the world’s finest athletes: periodic health evaluation practices of the top performing National Olympic Committees from the 2016 Rio or 2018 PyeongChang Olympic Games. Br J Sports Med. 2021;55(17):961–7. 33468453 10.1136/bjsports-2020-103481

[107] Boden BP, Osbahr DC. High- risk stress fractures: evaluation and treatment. J Am Acad Orthop Surg. 2000;8:344–53. 11104398 10.5435/00124635-200011000-00002

[108] Warden SJ, Edwards WB, Willy RW. Optimal load for managing low-risk tibial and metatarsal bone stress injuries in runners: the science behind the clinical reasoning. J Orthop Sports Phys Ther. 2021;51:322–30. 33962529 10.2519/jospt.2021.9982

[109] Arends JC, Cheung MY, Barrack MT, Nattiv A. Restoration of menses with nonpharmacologic therapy in college athletes with menstrual disturbances: a 5-year retrospective study. Int J Sport Nutr Exerc Metab. 2012;22:98–108. 22465870 10.1123/ijsnem.22.2.98

[110] De Souza MJ, Williams NI. Physiological aspects and clinical sequelae of energy deficiency and hypoestrogenism in exercising women. Hum Reprod Update. 2004;10:433–48. 15231760 10.1093/humupd/dmh033

[111] De Souza MJ, West SL, Jamal SA, Hawker GA, Gundberg CA, Williams NI. The presence of both an energy deficiency and estrogen deficiency exacerbate alterations of bone metabolism in exercising women. Bone. 2008;43:140–8. 18486582 10.1016/j.bone.2008.03.013

[112] Ihle R, Loucks AB. Dose-response relationships between energy availability and bone turnover in young exercising women. J Bone Miner Res. 2004;19:1231–40. 15231009 10.1359/JBMR.040410

[113] Misra M, Prabhakaran R, Miller KK, Goldstein MA, Mickley D, Clauss L, et al. Weight gain and restoration of menses as predictors of bone mineral density change in adolescent girls with anorexia nervosa-1. J Clin Endocrinol Metab. 2008;93:1231–7. 18089702 10.1210/jc.2007-1434 PMC2291495

[114] Fredericson M, Kent K. Normalization of bone density in a previously amenorrheic runner with osteoporosis. Med Sci Sports Exerc. 2005;37:1481–6. 16177598 10.1249/01.mss.0000177561.95201.8f

[115] Zanker CL, Cooke CB, Truscott JG, Oldroyd B, Jacobs HS. Annual changes of bone density over 12 years in an amenorrheic athlete. Med Sci Sports Exerc. 2004;36:137–42. 14707779 10.1249/01.MSS.0000106186.68674.2C

[116] Temme KE, Hoch AZ. Recognition and rehabilitation of the female athlete triad/tetrad: a multidisciplinary approach. Curr Sports Med Rep. 2013;12:190–9. 23669090 10.1249/JSR.0b013e318296190b

[117] Harmon KG. Lower extremity stress fractures. Clin J Sport Med. 2003;13:358–64. 14627867 10.1097/00042752-200311000-00004

[118] Ardern CL, Glasgow P, Schneiders A, Witvrouw E, Clarsen B, Cools A, et al. 2016 consensus statement on return to sport from the First World Congress in Sports Physical Therapy. Bern Br J Sports Med. 2016;50:853–64. 27226389 10.1136/bjsports-2016-096278

